# Indications for floaterectomy: results of a national BEAVRS survey

**DOI:** 10.1038/s41433-025-04087-4

**Published:** 2025-10-24

**Authors:** Varo Kirthi, Giampaolo Gini, Anthony G. Casswell, Edward H. Hughes, Edward J. Casswell

**Affiliations:** 1 Moorfields Eye Hospital, London, UK; 2 University Hospitals Sussex (Southlands Hospital), Worthing, UK; 3 University Hospitals Sussex (Sussex Eye Hospital), Brighton, UK; 4 University Hospitals Sussex (Southlands & Sussex Eye Hospitals), Worthing & Brighton, UK

**Keywords:** Health services, Outcomes research

Vitrectomy for floaters (‘floaterectomy’) in an otherwise healthy eye remains a contentious issue. Proponents argue that it is an underutilised effective intervention that improves quality of life, while others contend that it is an unnecessary procedure with underestimated risks.

Recent systematic review data suggest that whilst over 90% patients are satisfied with post-operative outcome, observed risks include cataract (1-in-3), retinal breaks (2–3%), retinal detachment (1.0–1.5%) and endophthalmitis (0.1–0.2%) [1]. Given the paucity of UK data on surgical indications and risks discussed with patients, a national survey was conducted to establish current practice among vitreoretinal surgeons in the United Kingdom.

An online questionnaire was sent to all BEAVRS members in the United Kingdom, and responses were collected between March and June 2024. Fourteen multiple-choice questions were asked on the management of floaters, in both National Health Service (NHS) and private healthcare settings. Data were gathered on procedures offered to patients in both settings and any criteria used prior to listing.

Of the 68 survey respondents, 62 (91.2%) reported working in the NHS. Only one (1.6%) offered laser vitreolysis on the NHS, whilst 53 (85.5%) offered floaterectomy. Among the latter, 48 (90.6%) ensured certain criteria were present prior to listing, including (i) significant impact on quality of life (100%), (ii) duration of symptoms (83.3%), (iii) floaters visible on clinical examination (75.0%), presence of a posterior vitreous detachment (37.5%) and floaters detectable on imaging (e.g., shadows on widefield fundus imaging, 22.9%). During consent, the most frequently quoted risk of reduced vision postoperatively was 1-in-100 (35.9%), followed by 1-in-1000 (25%), between 1–5% (23.4%) and >5% (1.6%). A small percentage of surgeons (4.7%) offered floaterectomy but did not quote a risk of reduced vision during consent.

Over 85% respondents offer floaterectomy on the NHS, with significant impact on quality of life being a ubiquitous criterion. Other criteria for surgery, including duration of symptoms, vary significantly. Of particular note, some surgeons may be underestimating risks when counselling; almost a third gave 0.1% risk of visual loss or did not state a risk, despite the published risks of retinal detachment (1.0–1.5%) and endophthalmitis (0.1–0.2%).
